# Is There A Role for Abscisic Acid, A Proven Anti-Inflammatory Agent, in the Treatment of Ischemic Retinopathies?

**DOI:** 10.3390/antiox8040104

**Published:** 2019-04-17

**Authors:** Pablo Baliño, Aurelio Gómez-Cadenas, Daniel López-Malo, Francisco Javier Romero, María Muriach

**Affiliations:** 1 Unitat predepartamental de Medicina, Universitat Jaume I, 12071 Castellón de la Plana, Spain; balino@uji.es; 2 Departament de Ciències Agràries i del Medi Natural, Universitat Jaume I, 12071 Castellón de la Plana, Spain; aurelio.gomez@uji.es; 3 Departamento de Ciencias Biomédicas, Universidad Europea de Valencia, 46010 Valencia, Spain; daniel.lopez2@universidadeuropea.es (D.L.-M.); franciscojavier.romero@universidadeuropea.es (F.J.R.); 4 Universitat Jaume I, Unitat predepartamental de Medicina, Avda/Sos Baynat, S/N, 12071 Castellón de la Plana, Spain

**Keywords:** abscisic acid, ischemic retinopathy, oxidative stress, angiogenesis, inflammation

## Abstract

Ischemic retinopathies (IRs) are the main cause of severe visual impairment and sight loss, and are characterized by loss of blood vessels, accompanied by hypoxia, and neovascularization. Actual therapies, based on anti-vascular endothelial growth factor (VEGF) strategies, antioxidants or anti-inflammatory therapies are only partially effective or show some adverse side effects. Abscisic acid (ABA) is a phytohormone present in vegetables and fruits that can be naturally supplied by the dietary intake and has been previously studied for its benefits to human health. It has been demonstrated that ABA plays a key role in glucose metabolism, inflammation, memory and tumor growth. This review focuses on a novel and promising role of ABA as a potential modulator of angiogenesis, oxidative status and inflammatory processes in the retina, which are the most predominant characteristics of the IRs. Thus, this nutraceutical compound might shed some light in new therapeutic strategies focused in the prevention or amelioration of IRs-derived pathologies.

## 1. Ischemic Retinopathies (IRs)

The retina, located at the back of the eyecup, is a multilayer tissue with the main purpose of performing phototransduction [1]. Neovascularization, or the formation of new blood vessels from the existing vasculature, probably after an ischemic event, is often associated with aberrant formation of immature vessels, and is one of the most common hallmarks of blinding diseases. Ischemic retinopathies (IRs), such as retinopathy of prematurity (ROP), diabetic retinopathy (DR), and age-related macular degeneration (AMD), are the main causes of severe visual impairment and sight loss in children, adults (with diabetes), and the elderly population, respectively [2]. Other ischemic ocular diseases are sickle cell retinopathy, retinal vein occlusion and several inflammatory diseases of the eye. However, the proportion of visual loss caused by these other diseases is much smaller.

IRs are biphasic diseases characterized by the loss of blood vessels, which is accompanied by hypoxia that in turn, induces a vasoproliferative phase in which aberrant immature blood vessels grow into the vitreous humor. These events can result in retinal detachment and vision loss [3]. It is also noteworthy that the ischemia associated with vessel loss can also impair neuronal function, together with the fact that retinal neurons secrete molecules in response to ischemia or stress, which are able to modulate vascular outgrowth [4].

In the case of ROP, in premature neonates, the retina remains incompletely vascularized at the time of birth and the vasculogenesis process in the premature neonatal retina becomes disrupted. Therefore, abnormal new proliferating vessels develop at the juncture of vascularized and avascular retina, growing from the retina into the vitreous. This phenomenon can result in hemorrhage and tractional detachment of the retina [5].

DR is the leading cause of blindness in adults of working age. The classification of diabetic retinopathy into stages is based on the presence of visible ophthalmologic changes and the manifestation of retinal neovascularization. The fourth stage is severe non-proliferative diabetic retinopathy and patients at this stage likely progress to the proliferative stage [6]. Proliferative diabetic retinopathy (PDR) refers to a severe stage of DR in which new vessels proliferate on the surface of the retina and posterior surface of the vitreous [7,8]. The duration of diabetes is clearly related to the development and progression of retinopathy and a good glycemic control reduces the progression of retinopathy [9,10]. Finally, serious visual loss in PDR is caused by vitreous hemorrhage and tractional retinal detachment.

AMD is the main cause of severe visual loss in the elderly and its prevalence increases every year due to the exponential aging of the population. Two types of AMD are clinically recognized: dry AMD which is characterized by the formation of extracellular deposits called drusen, followed by retinal pigment epithelium and photoreceptor cell death, and geographic atrophy and wet AMD with the presence of neovascularization. Contrary to ROP and PDR, in wet AMD neovascularization originates from the choroidal vasculature and extends into the subretinal space. This choroidal neovascularization takes place in the macula, the area of retina responsible for central vision, and in consequence it causes severe visual loss in AMD patients [11,12].

Neovascularization is characterized by the occupation of avascular areas with uncontrolled growing of blood vessels characterized for being sinuous, not well organized, and with tendency to exudation/leakage, fibrosis and cellular inflammation processes. Among many known growth factors, research has demonstrated that vascular endothelial growth factor (VEGF) is an endothelial cell-specific growth and survival factor and a significant factor responsible for vasculogenesis and neo-angiogenesis, both in physiological and pathological events. In this sense, VEGF seems to be responsible for the anomalous growth of blood vessels, the blood-retinal barrier (BRB) breakdown and the consequent vascular edema [13].

Among the factors involved in the pathogenesis of IRs, inflammatory processes and oxidative stress are crucial [2,14,15]. In addition, other factors involved in these pathologies, such as hyperoxia in ROP, hyperglycemia in DR, and lipid accumulation in AMD, are relevant amplifiers of oxidative stress causing cell metabolism dysregulation [16,17,18]. Furthermore, much evidence suggests that ROS may play a role in angiogenesis, due in part to the involvement of reactive oxygen species (ROS) in the mitogenic cascade initiated by the tyrosine kinase receptors of several growth factor peptides. Thus, Colavitti and coworkers, reported that VEGF utilizes ROS as messenger intermediates downstream of the VEGF receptor-2 (VEGFR-2)/KDR receptor [19]. Interestingly, oxidative stress and inflammation are tightly interconnected. Inflammation is a cellular response to different insults (among them oxidant stress) that compromises cell and tissue homeostasis, but also acts as a defense mechanism to preserve the stability of cellular functions. However, sustained inflammation can be detrimental to tissue integrity. Interestingly, it has been shown that the increased VEGF levels could be, at least in part, a consequence of an inflammatory environment characterized by the release of proinflammatory cytokines [20].

The retina is extremely rich in polyunsaturated lipids (such as docosahexaenoic acid, DHA), cis-arachidonic acid, and choline phosphoglyceride, and therefore it is very sensitive to oxygen and nitrogen reactive species and lipid peroxidation. In addition, it is characterized by a high-energy demand and an exposure to light; altogether, these conditions favor oxidative stress situations in this tissue. Antioxidant defenses, such as glutathione (GSH) or glutathione peroxidase (GPx) are compromised in DR [14] or AMD [21], as well as oxidative damage to macromolecule (such as lipids, DNA or proteins) increases in IRs [22,23]. The accumulation of peroxides likely induces thromboxane A2 production, a potent cytotoxic agent to microvasculature [24]. On the other hand, nitrosative stress can result in cis- to trans-isomerisation of arachidonic acid, which in turn has been shown to cause retinal vascular degeneration in a mouse model of ROP [25]. Platelet-activating factor and lysophosphatidic acid are other lipids generated during peroxidation that act as pro-inflammatory mediators and contribute to microvascular injury in the retina. Platelet-activating factor is profusely secreted under oxidative stress situations, and its cytotoxic effects are mediated, to a large extent, by thromboxane A2 [24]. Along the same lines, lysophosphatidic acid is released from lysophosphatidylcholine by the action of lysophospholipase D and can play a role in retinal inflammation, leading to microvascular cytotoxicity in oxygen-induced retinopathy (OIR) [26], thus connecting oxidative stress and inflammatory processes.

### 1.1. Possible Therapies for IRs

Anti-VEGF strategies, which include neutralization by engineered antibodies, chimeric receptors, or laser treatment that alleviates ischemia, an important stimulus for VEGF expression, have been proposed as promising therapies [15,27]. Although partially effective, several side adverse effects have been associated with anti-VEGF agents, including visual impairment since this growth factor has also been shown to have neuroprotective functions in the survival of retinal ganglion cells [28]. Therefore, advances in pharmacological antiangiogenic therapies are needed to improve the efficacy and safety of these agents. In addition, current therapies only target the advanced stages of IRs, more concretely the vasoproliferative phase and probably acting at the initial ischemic stage would better contribute to curtail the progression of these diseases.

As mentioned above, inflammation is a key factor in the development and progression of IRs. The use of corticosteroids, have been also approved for the clinical treatment of several IRs because of their ability to modulate inflammation-mediated neovascularization in the retina, being actually used as adjuvants in anti-VEGF therapies. However, clinical differences have been observed in efficacy, pharmacokinetics, and safety profiles, associated to each specific molecule administered, as well as inter-individual variation to treatment [3]. Furthermore, Ketorolac a nonsteroidal antiinflammatory drug that inhibits the synthesis of prostaglandins or anti-IL-1 treatments have been tried in ROP [2]. Other molecules such as compounds with anti-TNFα properties in DR, or mTOR inhibitors in AMD have been also proposed as anti-inflammatory therapies [2].

On the other hand, rather than developing new synthetic drugs that non-selectively target angiogenic and non-angiogenic signaling pathways, evaluating natural phytochemicals, which generally act through multiple cell-signaling mechanisms, but minimally affect the overall health of tissues, seems a better alternative approach. Since during retinal ischemia the imbalance between the production of ROS and the ability to scavenge these ROS is disturbed, ROS are able to trigger several signaling pathways and affect DNA, proteins and lipids inside the cell, leading to cell death. Therefore, antioxidants can protect retinal cells from microvascular degeneration in IRs, and in fact supplementation with different compounds such as vitamin E, vitamin C, lipoic acid or different polyphenols (i.e., resveratrol) and carotenoids (among them, lutein and zeaxanthin, the main macular pigments) have shown promising effects for the treatment of these diseases [2,14,22].

Abscisic acid (ABA), a phytohormone most commonly known for inhibiting germination, could be also an example of such a natural product with novel properties to explore in the treatment of inflammation-induced vasoproliferative disorders.

### 1.2. Phytohormones are Cross-Kingdom Molecules

Plants have developed different, well-conserved evolutive mechanisms which allow them to overcome multiple environmental conditions such as water or nutrient availability, ultraviolet light, temperature variations, parasite threatens, etc. Most of these responses are mediated by plant hormones or phytohormones. Particularly, these molecules are a diverse group of natural metabolites with a low molecular weight that act at micromolar or even lower concentrations [29]. Phytohormones have been proved to control the different plant developmental stages and are responsible for the mechanisms that trigger responses to adverse environmental conditions, which are essential to improve or achieve stress tolerance [30].

There are different groups of phytohormones that notably vary in molecular structure and functional properties being named as ABA, gibberellins, cytokinins, ethylene, auxins, jasmonates, salicylic acid, and the recently discovered brassinosteroids and strigolactones [29]. Despite their known functional role on plants, phytohormones have proved to have a function on organisms from other kingdoms. Thus, animals, including humans, can sense and produce plant hormones [31,32].

## 2. Abscisic Acid (ABA)

Chemically, ABA has a formula C15H20O4, and its appearance is colorless crystals. ABA belongs to the terpenoid (also known as isoprenoid) class of plant metabolites. The ABA molecular structure have some peculiarities worth to note, such as the side chain with two double bonds conjugated to the carboxylic acid; with the closest double bond to the acid group being cis, and the one placed closer to the ring being trans. Under ultraviolet (UV) radiation this configuration can be reversibly changed to the inactive form 2-4-trans [33]. On its structure, there is an asymmetric carbon on position 1’ that provides specificity for the described receptors in plants [34].

In animal tissues, the presence of ABA has been known since the early 1980s but unfortunately ignored until the last decade. Recently, ABA has emerged as a key modulator of different human physiological processes.

ABA is commonly named the ‘stress hormone’ and regulates many aspects of plant growth and development [35,36]. Under stressful situations, ABA induces a burst of the plant’s antioxidant defense system. Also, it seems to inhibit germination and promoting plant dormancy. ABA was discovered in the 1960’s and due to its function on plant dormancy was called “Dormidin”. At the same time, other researchers described ABA’s properties on fruit abscission, naming it as ‘Abscisin II’ [33]. However, a later accurate identification of this substance revealed that both functions were carried out by the same molecule and after deliberations ABA was chosen as the most suitable name. Since then, biochemical, molecular and genomic approaches were developed with the aim of elucidating the ABA biosynthetic and catabolic pathways, identifying any possible ABA transporters and shedding some light into the complex signaling components associated with the ABA response [37]. Moreover, the discovery of the ABA receptors and other genes involved in ABA downstream signaling cascade constituted an important milestone that deepened the understanding of the ABA mode of action [38].

## 3. ABA Actions in Animals

In animals, ABA naturally originates from different dietary sources but also is endogenously produced by the carotenoid biosynthesis pathway

In mammals, the best proposed mechanism for ABA actions is the G-coupled membrane protein called lanthioninesynthetase C-like protein 2 (LANCL-2) [39,40]. This hormone binding to LANCL-2 triggers a PKA-dependent cascade, which activates the ADP-ribosyl cyclase, mobilizes cyclic ADP-ribose and leads to an increase on cyclic AMP-dependent cellular calcium. This phytohormone, can be administered either through different nutritional sources or as a drug. In fact, it has been proved that in mice, high doses of ABA can be tolerated without any side effect [41]. Moreover, there is also endogenous production of ABA in many different cell types such as granulocytes, macrophages, keratinocytes, microglia and stem cells [42].

At the functional level, it has been demonstrated that ABA plays a key role in resistance against different microbial pathogens [43,44,45]. Zhou and coworkers, 2016; studied ABA effects on brain gliomas. This data support a key role of ABA in promoting apoptosis of cancer cells [46]. Moreover, a protective role on type 2 diabetes has been demonstrated [47,48]. On this respect, plasmatic ABA (ABAp) levels, correlate with different glucose dysregulation conditions. For example, the hyperglycemia observed in T2D and in gestational diabetes (GDM) is also accompanied by an increase of ABAp levels. In the case of GDM, there is a link between spontaneous remission (after childbirth) and a restoration of a normal ABA response to oral glucose [48]. A similar link is observed in T2D patients, in which resolution of diabetes was observed after biliopancreatic diversion (BPD). In this case, pre-BPD values and basal ABAp were, compared to the latter, significantly increased 1 month after BPD in T2D as well as in normal glucose tolerant subjects, in parallel with a reduction of fasting plasma glucose [47]. These results highlight the critical role of ABA in mediating the normal and pathogenic glucose tolerance levels concluding that ABA can improve glucose tolerance.

Furthermore, at a central level, ABA administration in a mice model has demonstrated an anti-inflammatory protective effect, lowering microglia activation, decreasing TNFα levels, and restoring high-fat diet induced cognitive dysfunctions [49]. Recent studies also support an ABA role in modulating hippocampal neurogenesis [50].

Another studied action of this phytohormone are its role on the inflammatory response. It has been described that ABA plays a dual function since has pro- and anti-inflammatory actions. In this respect, two differential signaling pathways have been described as key modulators of these two inflammatory opposite responses. Thus, ABA’s pro-inflamatory response is mediated by macrophage G protein-coupled receptors (GPCRs). These receptors are expressed in the immune system cells and trigger the production of cytokines, and other pro-inflammatory molecules [51]. On the other hand, ABA actions through the LANCL2-PPARγ cascade have been linked to its anti-inflamatorry effects. The peroxisome proliferator activated receptors (PPARs) are a subset of the nuclear receptor superfamily [52]. Particularly, PPARγ is expressed in a wide range of tissues including immune cells. Its anti-inflammatory effects are mediated by a repression of the macrophage pro-inflammatory genes [53]. However, as endogenous synthetized anti-inflammatory molecule, very little is known about the human function and regulation of ABA.

As mentioned, in plants ABA is a ubiquitous hormone, which modulates critical plant processes such as germination and development and regulates stressful situations. However, ABA has also been studied for its properties as a potent antioxidant molecule. Under stress conditions, ABA triggers a potent burst on the plant antioxidant defense system increasing the activity of superoxide dismutase, catalase, peroxidase, and glutathione reductase [54]. Moreover, ABA can elicit a response on gene expression to increase the activity of these antioxidant enzymes [54,55,56]. In animals, it has been recently described an antioxidant protective effect of ABA. Rafiepour and coworkers [57] studied ABA neuroprotective properties against 6-OHDA-induced neurotoxicity on an in vitro model of Parkinson’s disease. They demonstrated ABA’s antioxidant (by reducing ROS levels) and antiapoptotic properties mediated by the PPARγ signaling cascade [57]. Furthermore, Soti and coworkers have recently reported that ABA is also able to reduce oxidative stress in rat brain [58]. Briefly, the central microinjection of ABA was able to reduce MDA concentration, H2O2 levels in rat diencephalon as well as to increase the antioxidant enzymes catalase and peroxidase activities [58].

## 4. ABA Presence in Food and Its Relationship with Lutein and Zeaxanthin

**Figure 1:**
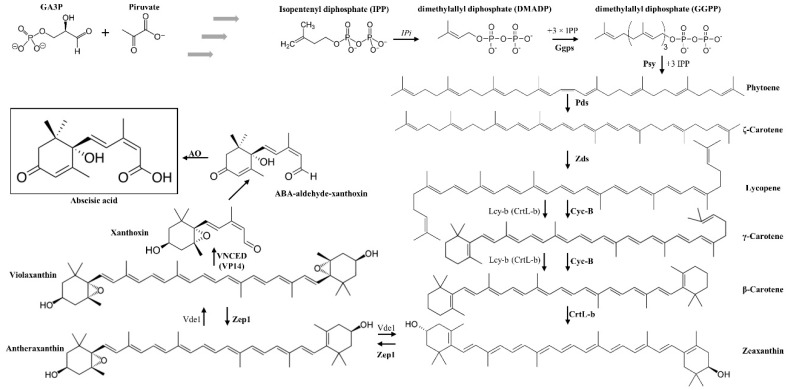
Abscisic acid (ABA) biosynthesis pathway in plants: Ggps: Geranylgeranyl pyrophosphate synthase, Pds: 15-cis-phytoene desaturases, VNCED (VP14): 9-cis-epoxycarotenoid dioxygenase, Psy: phytoene synthase, Zds: zeta-carotene desaturase, AO, aldehydes oxidase, LCY-B/CRTL-B: Lycopene beta-cyclase, Zep1: Zeaxanthin epoxidase1, IPi: IPP isomerase, Vde1: Violaxanthin de-epoxidase, lycopene β-cyclase, CrtR-b: β-ring hydroxylase [65].

As mentioned above, ABA belongs to the terpenoid class of plant metabolites. ABA biosynthesis is derived from C40 epoxycarotenoid precursor through an oxidative cleavage reaction in plastids, what is known as the indirect or C40 pathway in plants. This route is started from isopentenyl pyrophosphate (IPP), a C5 terpenoid precursor [59,60]. IPP is converted into geranylgeranyl pyrophosphate (GGPP) through the action of IPP isomerase and GGPP synthase enzymes [61]. A series of intermediate reactions occur but the first step more specific to the ABA synthesis pathway is the epoxidation of zeaxanthin and antheraxanthin to violaxanthin. This precursor violaxanthin is converted to xanthoxin, which is then exported to the cytosol. Here, a two-step reaction occurs and the product xanthoxin is converted to ABA (Figure 1).

In this biosynthetic pathway, two carotenoids are mainly produced, lutein and zeaxanthin. In this respect, previous works support the beneficial effects of these molecules against eye diseases, being critical in the prevention of age-related macular degeneration [62,63].

Although the absence of reports that analyze the concentrations of lutein and zeaxanthin jointly with ABA, some works reveal that, the nutraceutical role of ABA could be similar to the mentioned for lutein and zeaxanthin [64]. In fact, several vegetables such as maize, kiwi, red grapes, zucchini squash, etc. have been proposed as food with high contents of lutein and zeaxanthin [64]. Other vegetables have been reported to have high contents of ABA, including avocado, citrus, soybean or maize [64]. Although some of the analyzed vegetables such as corn, had high concentrations of lutein, zeaxanthin and ABA simultaneously, it seems that there is not a direct correlation between them [63,64]. Moreover, in non-vegetable edibles, egg yolk has been also reported as a food rich in lutein and zeaxanthin [64].

## 5. ABA as a Novel Therapy for IRs

To date, the majority of the evidence on protective effects ABA in human health has addressed glucose metabolism and anti-tumorigenic effects. In this review, we describe various aspects of ABA mechanisms of action and discuss the potential role of this phytohormone in the treatment of the most common neovascular retinopathies. At the moment, there is almost no literature about the direct functional effects of ABA in this neurosensorial tissue. However recently, Chaqour and coworkers have studied the effects of ABA administration on physiological and pathological angiogenesis processes in the retina, particularly in a mouse model of OIR [27]. Interestingly, they showed that this phytohormone acts largely by altering the phenotypical plasticity of endothelial cells and skewing the canonical polarized inflammatory statuses of macrophages towards an antiangiogenic phenotype. This mechanism is based on ABA suppression of the neo- and revascularization processes of the retina by knocking down endogenous pro-migratory genes in endothelial cells, thus promoting endothelial quiescence.

Moreover, they also examined if ABA was able to modulate macrophage recruitment and polarization during retinal neovascularization. Although ABA did not affect macrophage infiltration of the ischemic areas in the retina, the treatment with this phytohormone significantly increased the amount of OIR-induced anti-angiogenic macrophage markers and dampened pro-angiogenic macrophage-related markers. It is especially noteworthy that ABA also reduced significantly VEGF expression in the retina, which is the major growth factor involved in angiogenesis [27].

**Figure 2:**
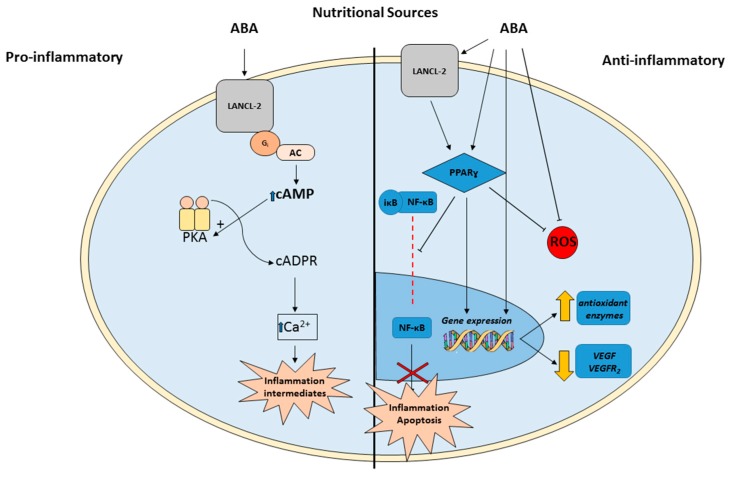
Schematic representation of the pro- and anti-inflammatory pathways mediated by ABA in animals with special focus on a *potential* anti-inflammatory, antioxidant and anti-angiogenic retinal mechanism of action. cAMP: Cyclic adenosine monophosphate; AC: Adenylyl Cyclase; PKA: Protein Kinase A; PPARγ: Peroxisome Proliferator-Activated Receptor Gamma; NF-κB: Nuclear Factor- Kappa B; VEGF: anti-vascular endothelial growth factor; LANCL-2: lanthioninesynthetase C-like protein 2; ROS: Reactive Oxygen Species.

As mentioned above, inflammatory processes and oxidative stress are also crucial factors in the pathogenesis of IRs and, although there is no literature about the direct effects of ABA in the retina, this hormone is able to modulate cell oxidative status by enhancing the antioxidant defense in plants and in different animal tissues [57,58,66]. In addition, ABA has also revealed prominent anti-inflammatory properties [67]. Thus, ABA upregulates the PPARγ both in vitro and in vivo [45] inhibiting among others, NFkB translocation to the nucleus [68]. Both of them, PPARγ, and NFkB are transcription factors expressed in retinal tissue able to modulate oxidative stress situations [14,69]. In addition, PPARγ agonists have protective effects against oxidative damage, inducing the expression of antioxidant enzymes such as catalase or superoxide dismutase [70]. Moreover, there is evidence that PPARγ might be involved in the mechanisms underlying angiogenesis regulation. It has been demonstrated that PPARγ is able to reduce the VEGFR-2 expression in a mouse model of IRs, thus inhibiting neovascularization in this tissue [69]. On the other hand, NFkB, which is blocked by PPARγ, is a sensor of oxidative stress. ROS activate this transcription factor, which, in turn, translocates from the cytosol into the nucleus and subsequently activates a variety of target genes related to inflammatory processes and apoptosis. These genes have proved to be involved in the development and progression of IRs [14,71] (Figure 2). Moreover, different data support that retinal NFκB transcriptional activity plays a pivotal role modulating ischemia-independent mechanisms, which lead to local activation of angiogenic cascades, showing an interesting link between VEGF and NFκB [72].

It is noteworthy to remark that ABA has been reported to play an important role in the maintenance of glycemic control [73]. As mentioned above, a good glycemic control reduces the progression of retinopathy [9,10], thus ABA appears to be a promising therapy for DR.

## 6. Conclusions

It is importantly to highlight that ABA’s anti-angiogenesis properties have not been studied in detail yet. However, this phytohormone present in vegetables and fruits that can be naturally supplied by the dietary intake has previously shown other benefits for human health in terms of glucose metabolism, inflammation and oxidative stress, all of them factors that are involved in IRs development and progression. This review focuses on a novel and promising role of ABA as a modulator of angiogenesis, which is the most relevant feature in IRs. Considering that, the actual therapies for these pathologies are only partially effective and show adverse side effects, these ABA properties might shed light on the potential therapeutic role to prevent or ameliorate IRs. Further experiments are necessary to determine the presence of ABA in the retina, and to elucidate its promising beneficial effect as an anti-inflammatory and antioxidant compound for the treatment of IRs.
